# Where does the bias begin? Cognitive processing differences in the aesthetic evaluation of AI-generated art

**DOI:** 10.3389/fpsyg.2026.1827956

**Published:** 2026-06-03

**Authors:** Yongquan Wang, Yiwei Cao, Jingnan Cai, Bin Sun

**Affiliations:** 1 School of Theater, Film and Television, Communication University of China, Beijing, China; 2 School of Mechanical Engineering, University of Science and Technology Beijing, Beijing, China

**Keywords:** aesthetics, artificial intelligence, computer-generated art, dual-process theories, meaning construction

## Abstract

Drawing on the Pleasure-Interest Model of Aesthetic Liking (PIA model), we investigated whether the negative bias toward AI-generated art arises primarily during perceiver-driven controlled processing rather than stimulus-driven automatic processing. Across three experiments in which all presented artworks were exclusively AI-generated while only the creator labels were manipulated, we showed that this bias does not primarily stem from rapid, stimulus-driven automatic processing. Instead, it emerges predominantly during controlled processing. The results indicated that the negative bias was markedly smaller under conditions favoring automatic processing than under conditions inducing controlled processing. Moreover, providing interpretive semantic cues—brief textual descriptions that support meaning construction during controlled processing—significantly mitigated the bias against AI-generated art.

## 1 Introduction

In recent years, the exponential growth of generative artificial intelligence (Generative AI) has fundamentally reshaped the landscape of creative production (Epstein et al., 2023). Although blind evaluations show that AI artworks possess objective artistic value (Di Dio et al., 2023; Cenerini et al., 2025), audiences still display strong negative es toward them (Hong, 2018; Ragot et al., 2020). This tension between objectively high quality and subjective devaluation highlights a critical challenge. Understanding the psychological roots of this bias and identifying effective interventions have become urgent issues in human-computer interaction and empirical aesthetics (de Rooij, 2025).

Why do humans develop biases against AI-generated art? Prior studies suggest that many viewers experience negative emotions toward AI artworks because they believe AI lacks the emotional depth and creativity essential to human artistic creation (Ragot et al., 2020). Individual differences also play an important role. Personality traits and prior experiences shape attitudes toward AI art. For example, unfamiliarity with technology or previous negative encounters can lead to stronger prejudice (Grassini and Koivisto, 2024). In addition, bias against AI art may stem from anthropocentric beliefs. People often assume that only human artists can produce works with genuine emotion and creativity. This assumption makes human art appear more valuable by comparison (Millet et al., 2023). Although existing research has identified these psychological factors, a unified mechanistic framework is still lacking. Such a framework is needed to explain these phenomena and to guide effective strategies for reducing bias against AI art (Zhang et al., 2022).

A common methodological approach in this line of research is the “creator-label manipulation” paradigm, in which identical or comparable artworks are presented under different source labels (AI-generated vs. human-created) while aesthetic evaluations are recorded. This paradigm has been adopted by multiple research teams to demonstrate the existence of anti-AI bias (Chamberlain et al., 2018; Hong and Curran, 2019; Ragot et al., 2020; Gangadharbatla, 2022; Bellaiche et al., 2023; Di Dio et al., 2023; Millet et al., 2023). Although these studies have consistently confirmed a negative labeling effect, several methodological limitations remain unresolved and constrain the interpretability of their findings. First, most studies have used generated images directly as stimuli without systematic perceptual screening; identifiable artifacts in AI images (e.g., distorted hands, unnatural textures) may serve as confounding cues that drive evaluations independently of the label itself (Chamberlain et al., 2018; Ragot et al., 2020). Second, stimulus sets in prior work have often been unbalanced with respect to artistic style, despite recent meta-analytic evidence that representational format (abstract vs. figurative) significantly moderates the magnitude of anti-AI bias (Di Dio et al., 2023; de Rooij, 2025). Third, low-level visual fluency features—which constitute the core antecedents of aesthetic preference within the PIA framework—have rarely been objectively quantified or controlled across stimulus sets (Reber et al., 2004; Graf and Landwehr, 2015). These limitations highlight the need for a more rigorously controlled investigation. The present study addresses these gaps through three methodological refinements: (1) a Two-Alternative Forced Choice (2AFC) pretest serving as a perceptual “Turing test” to retain only AI images that participants cannot reliably identify above chance; (2) balanced representation of abstract and figurative styles; and (3) objective quantification and control of nine visual fluency features using the Aesthetics-Toolbox (Mayer and Landwehr, 2018).

To examine the psychological mechanisms underlying this phenomenon, this study adopts the Pleasure-Interest Model of Aesthetic Liking (PIA model) proposed by Graf and Landwehr (2015) as its core theoretical framework. The PIA model integrates fluency theory and dual-process theory. It conceptualizes the formation of aesthetic preference as a two-level process:

(1) Automatic processing and aesthetic pleasure

This is the first stage of aesthetic experience. It is mandatory and primarily stimulus-driven. At this stage, if an aesthetic object (e.g., an AI-generated image) displays clear, symmetrical, or prototypical visual features, viewers experience higher processing fluency. This fluency elicits positive affective responses, which are then translated into aesthetic pleasure.

(2) Controlled processing and aesthetic interest

When viewers possess sufficient need for cognitive enrichment, aesthetic experience moves into a second stage. This stage is perceiver-driven and involves deeper processing, including active interpretation, meaning construction, and reflection. A key insight of the PIA model is that positive experience at this stage aesthetic interest does not depend on the absolute level of fluency. Instead, it depends on disfluency reduction. Through cognitive effort, viewers successfully grasp complex intentions or resolve cognitive conflict. This process produces a dynamic increase in fluency and leads to deeper aesthetic satisfaction.

**Figure 1:**
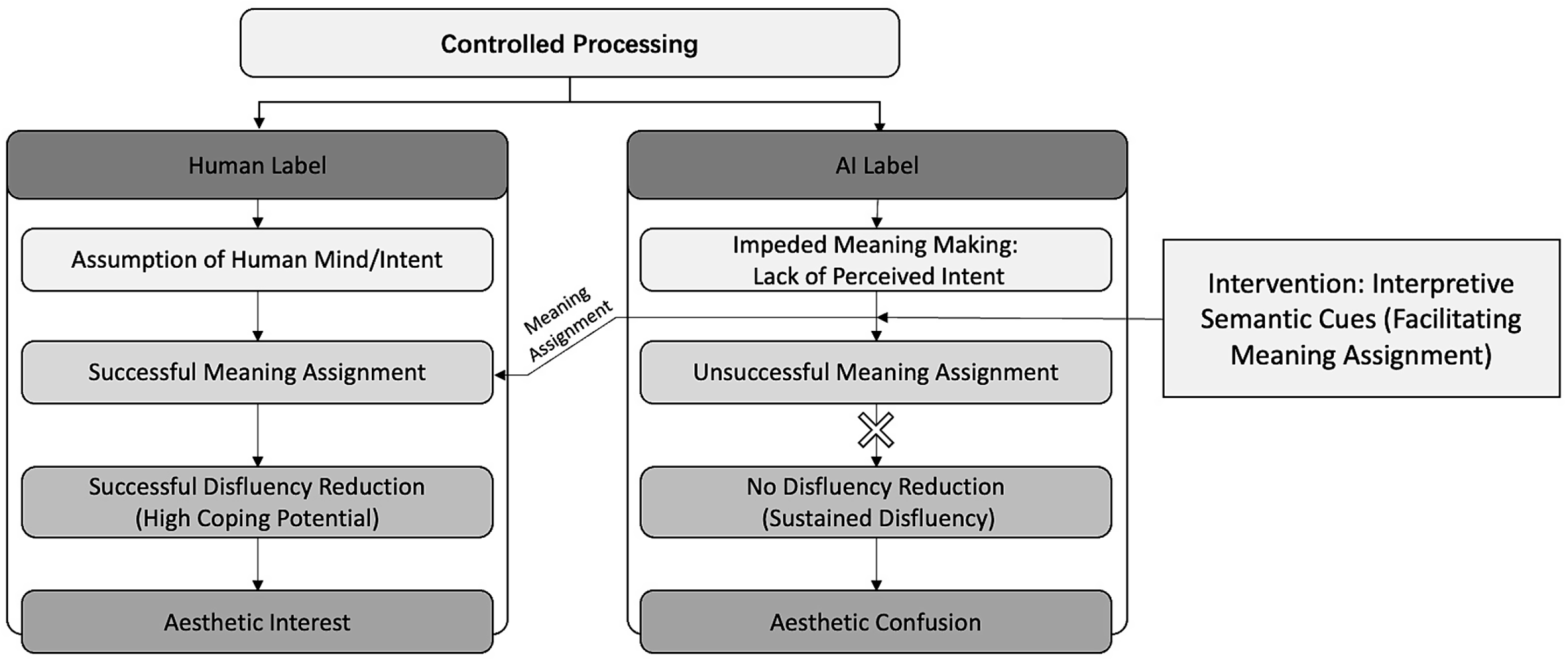
The conceptual framework based on the PIA model. Flowchart illustrating the hypothesized cognitive mechanisms during controlled processing. The “Human Label” facilitates successful meaning assignment and disfluency reduction, leading to aesthetic interest, whereas the “AI Label” impedes meaning making and results in aesthetic confusion. Interpretive semantic cues serve as an intervention to facilitate meaning assignment.

Based on the PIA model, this study proposes that anti-AI bias does not primarily arise during the initial stage of automatic processing. Instead, it emerges during the stage of controlled processing, where viewers engage in deeper cognitive elaboration and meaning construction. We hypothesize that the label “AI-generated” disrupts this controlled processing stage, leading to lower aesthetic evaluations. One plausible account is that this disruption occurs because the AI label undermines viewers’ expectations of creative intention, thereby impeding disfluency reduction. We further hypothesize that providing semantic cues that support meaning construction during controlled processing can alleviate this disruption and attenuate bias against AI-generated art (see Figure 1).

Although no empirical studies have yet systematically applied the Pleasure-Interest Model (PIA model) to explain bias against AI art, a growing body of recent findings on the acceptance of AI-generated art shows a strong structural alignment with the model’s theoretical framework.

Prior research indicates that decontextualized presentation often leaves viewers in a state of cognitive impasse, whereas the introduction of external information can substantially improve evaluations. This pattern directly reflects the PIA model’s emphasis on the controlled processing stage and its reliance on meaning assignment. For example, Mehler et al. (2024) found that evaluations increase significantly when viewers perceive human effort and involvement in the creative process, an effect commonly described as the “IKEA effect.” From a PIA perspective, information about human input functions as a critical form of contextual support for meaning construction—that is, external information that facilitates cognitive elaboration during the controlled processing stage. In addition, the meta-analysis by de Rooij (2025) shows that physical display contexts, such as gallery settings, can effectively attenuate anti-AI bias. Zhang and Li (2024) further demonstrate that helping viewers understand the AI workflow shifts their attitudes from resistance to acceptance. Together, these findings strongly support the PIA-based account proposed in this study. Bias does not arise solely from low-level automatic processing. Instead, it is largely driven by the absence of supportive contextual information that guides cognitive elaboration. When contextual cues about the creative process or presentation environment are provided, viewers’ controlled processing is better supported, which in turn alleviates bias against AI-generated art.

However, it is important to note that resistance to AI art does not always stem from failures in cognitive processing. In contemporary cultural discourse, skepticism toward AI art is often grounded in legitimate ethical and social concerns. These include the risk of displacing artistic labor (Jiang et al., 2023), issues of copyright and consent in training data (Samuelson, 2023), and the concentration of power within algorithmic systems of cultural production (Epstein et al., 2023). From this perspective, some forms of devaluation are not the result of aesthetic confusion or misunderstanding. Rather, they reflect normative value judgments. The present study does not deny the validity of these ethical positions. Instead, it focuses on a different analytical level: how aesthetic evaluations change at the cognitive level when ethical controversies are not explicitly activated.

The central theoretical contribution of this study lies in demonstrating that AI bias emerges primarily during the perceiver-driven controlled processing stage of the PIA model, rather than during automatic processing. We further explore whether providing contextual support during this stage can attenuate the bias. Value blindness arising from habitual cognitive schemas not only obscures the true potential of this technology, but also constitutes a key bottleneck for the development of human-AI collaborative art. Through three sequential experiments, this research systematically tests the applicability of the PIA model to evaluations of AI-generated art. We aim to demonstrate that improving acceptance of AI art does not primarily depend on further enhancing visual-level “pleasure.” Instead, it requires reconstructing viewers’ “interest” through stronger contextual support. Based on this theoretical reasoning, the study proposes the following core hypotheses:

*H1:* When the same artwork is labeled as AI-generated (rather than human-created), people will exhibit significantly lower levels of aesthetic liking.

*H2:* Negative bias toward AI art is moderated by processing mode. The bias is significantly larger under perceiver-driven controlled processing than under stimulus-driven automatic processing.

*H3:* Providing interpretive semantic cues that support meaning construction during controlled processing (relative to no such cues) significantly reduces negative bias toward AI-generated (relative to human-created) artworks.

## 2 Overview of studies

This research systematically investigates the mechanisms underlying bias against AI-generated art and potential intervention strategies through three sequential experiments. Across all studies, participants viewed a series of paintings. Creator identity labels (AI-generated vs. human-created) were manipulated using a counterbalanced order. This design allowed us to measure overall aesthetic preference (Studies 1–3), as well as the specific components of aesthetic pleasure and aesthetic interest (Study 3). Study 1 first establishes the presence of a robust negative bias against AI art. The results confirm that this evaluative downgrade is independent of the artworks’ visual content. Study 2 adopts the dual-process perspective of the PIA model. By separating stimulus-driven automatic processing from perceiver-driven controlled processing, it examines at which stage of aesthetic evaluation the bias against AI art primarily emerges. Finally, Study 3 develops and tests a serial mediation model. By providing interpretive semantic cues that support meaning construction, we significantly reduced the liking gap between AI-labeled and human-labeled artworks (Study 3, condition × label interaction on liking, *p* < 0.001). Mediation analyses further revealed that this reduction operated specifically through enhanced aesthetic interest—the hallmark of successful disfluency reduction during controlled processing—rather than through immediate aesthetic pleasure. This dissociation provides converging support for the PIA-based account: the malleability of anti-AI bias lies in the interest pathway, not the pleasure pathway.

All studies complied with the ethical guidelines for research with human participants set by the American Psychological Association (APA). In each study, participants provided informed consent prior to voluntary participation. They received a fixed monetary compensation for their time and were free to withdraw at any point during the experiment. All experimental data were collected from independent samples. Because the studies involved manipulation of creator identity labels, post-experimental debriefing was conducted to ensure methodological rigor and ethical compliance. Participants were fully informed of the true purpose of the study and the necessity of the experimental manipulations. Only after participants confirmed their understanding and renewed consent were their data included in the final analyses. All measures, manipulations, and potential data exclusion criteria are reported transparently.

## 3 Stimulus materials

All stimuli used in the formal experiments (Studies 1–3) were AI-generated images. This design choice—using exclusively AI-generated artworks with manipulated creator labels—was deliberate and theoretically motivated. By holding visual content constant while varying only the source attribution, we could isolate the pure effect of the “AI-generated” label on aesthetic evaluation, eliminating potential confounds from actual quality differences between human and AI artworks.

To ensure these AI artworks were visually indistinguishable from human-created works and free from obvious technical artifacts, we conducted a rigorous two-stage selection process: initial generation followed by empirical screening through a pretest.

### 3.1 Initial image generation

We generated 200 paintings using the state-of-the-art image generation model Gemini 3 Pro Image. The initial stimulus pool covered a wide range of artistic styles and subject matters. This diversity ensured that the final stimulus set would be representative of various aesthetic dimensions and would not be confounded by a single stylistic category.

### 3.2 Pretest screening procedure

To validate that our AI-generated images could pass as human-created works, we conducted a visual discrimination pretest using a Two-Alternative Forced Choice (2AFC) paradigm (see Section 4 for detailed methods). For this screening task only, we compiled an additional comparison set of 200 human-created paintings from publicly available art databases, matched to the AI-generated images in style diversity and subject matter.

#### 3.2.1 Critical design note

Critical design note: The human-created paintings served exclusively as comparison stimuli during the pretest screening phase. None of these human-created images appeared in any of the three formal experiments. In all formal experimental conditions, creator identity (AI-generated vs. human-created) was manipulated solely through labeling, ensuring that all observed effects in the main studies can be attributed purely to the manipulated creator label rather than to any inherent visual differences between actual human and AI artworks.

**Figure 2:**
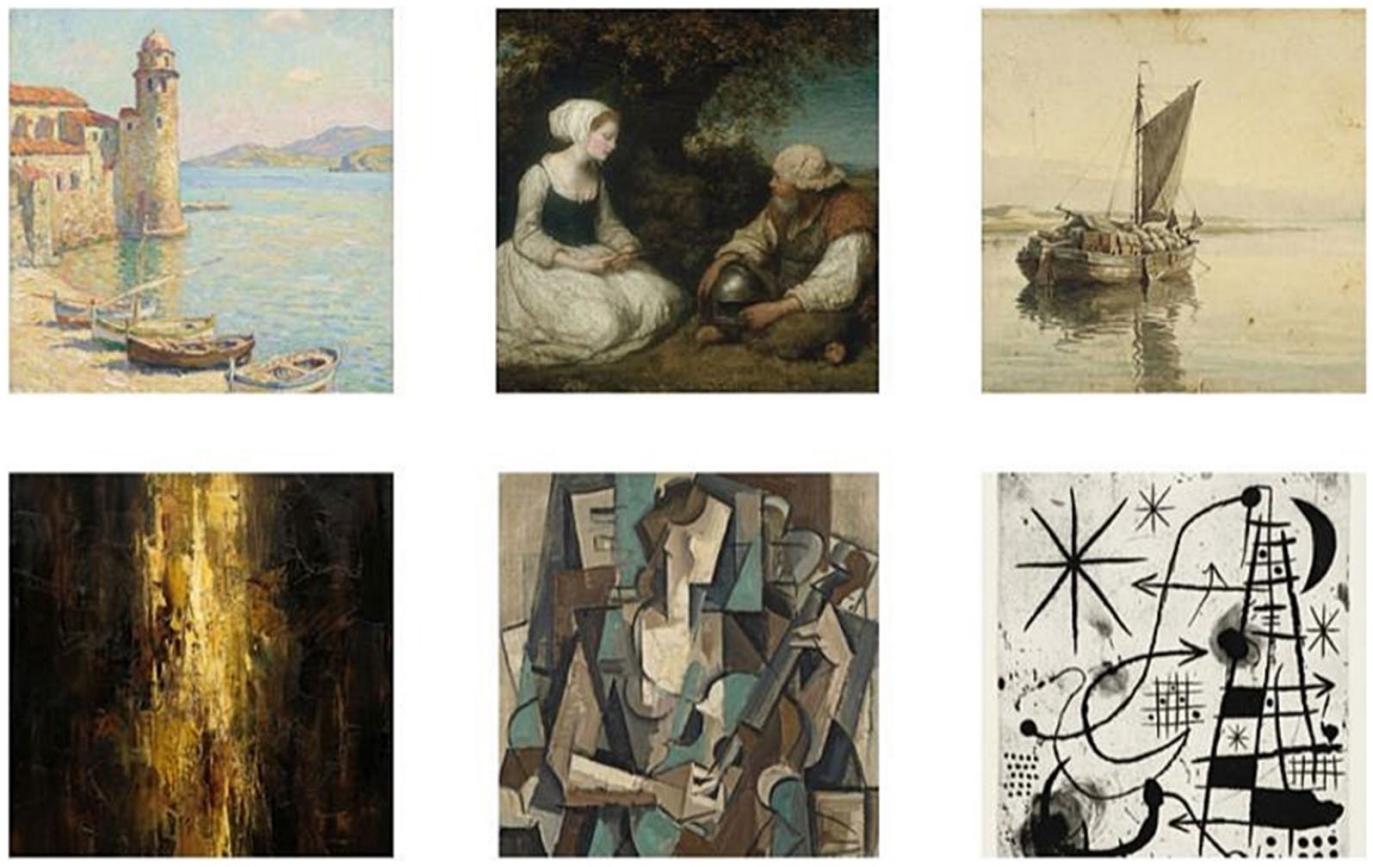
Examples of stimuli used in the experiments. A selection of AI-generated paintings used in Studies 1–3, covering both figurative (top row) and abstract (bottom row) styles. All experimental stimuli were exclusively AI-generated; only the creator labels were manipulated.

Based on the pretest results, we selected 94 AI-generated paintings that participants could not reliably identify as AI-generated at rates significantly above chance (see Section 4.2 for statistical details). These 94 images formed the core stimulus set for Studies 1, 2, and 3 (see Figure 2).

### 3.3 Stimulus balancing and control

Prior research has shown that the representational format of artworks (abstract vs. figurative) influences bias toward AI art (de Rooij, 2025). To ensure that variation in the dependent variables primarily resulted from the experimental manipulations rather than systematic differences in representational style, the final 94-image stimulus set was balanced to include equal numbers of abstract and figurative works.

Additionally, to control for the influence of visual fluency, we used the Aesthetics-Toolbox (Mayer and Landwehr, 2018) to extract nine objective features related to processing fluency: complexity, edge density, RMS contrast, mirror symmetry, balance, fractal dimension, self-similarity, edge-orientation entropy, and homogeneity. These features cover the four core dimensions of processing fluency proposed by Mayer and Landwehr (2018). All nine features were standardized and then aligned and weighted according to whether each feature facilitated or impeded fluency, yielding a composite Visual Fluency Score for each image.

Based on this index, images were classified into three fluency levels (high, medium, and low) using a tertile split. This balancing procedure was implemented at the stimulus-selection stage to ensure that no single fluency level disproportionately influenced the results. In subsequent studies (Studies 1–3), images from the three fluency levels were presented in approximately equal proportions.

### 3.4 Stimulus set size across studies

The 94-image stimulus pool was determined *a priori* to provide three non-overlapping subsets for the sequential experiments. Studies 1, 2, and 3 used 40, 30, and 24 paintings, respectively. These specific sample sizes were determined by balancing statistical power with participant burden and task-specific constraints. Study 1, which required only simple preference ratings, used a larger set to maximize measurement reliability. Study 2 maintained a moderate set size to accommodate the dual-task paradigm in the automatic processing condition, where participants needed to simultaneously maintain a four-digit number in working memory while viewing each painting. Study 3 employed the smallest set because participants needed to read curatorial descriptions (approximately 30 words each) for each artwork before rating, substantially increasing both trial duration and cognitive load.

## 4 Pretest

Before conducting the main experiments, we ran a pretest aimed at selecting AI-generated paintings that were visually difficult to distinguish from human-created works. This screening ensured strict control over visual quality as a confounding variable, allowing observed differences in the main experiments to be attributed solely to the creator label (AI vs. human) rather than perceptible flaws or artifacts in the artworks themselves.

### 4.1 Method

Over 240 participants were recruited for the pretest via convenience sampling across a university campus. To ensure each painting received sufficient evaluation, the 400 candidate images (200 AI-generated, 200 human-created) were divided into four groups, with at least 60 participants randomly assigned to each group. Sensitivity power analysis for one-sample t-tests was conducted using G*Power. With an alpha level of 0.05 and power of 80%, the sample size of 60 per group allowed detection of a minimum effect size of Cohen’s *d* = 0.37.

The pretest used a Two-Alternative Forced Choice (2AFC) paradigm. Each participant completed 50 trials. In every trial, one AI-generated painting and one human-created painting were presented side by side in random left–right positions. Participants were instructed to carefully examine the images and select the one they believed was AI-generated. To encourage careful responses and ensure data reliability, participants were informed that their compensation would depend on the accuracy of their judgments.

### 4.2 Results

We calculated the probability of each AI-generated painting being correctly identified as AI and compared it to the 50% chance level using one-sample t-tests. The results showed that most AI paintings were not recognized at rates significantly different from random guessing.

To meet the requirements of the three planned experiments, we applied a dual-criterion selection process: First, we retained only AI-generated paintings whose identification rates did not significantly exceed 50% (*p* > 0.05 in one-sample t-tests against chance). Second, from this qualified pool, we systematically selected 94 images that collectively satisfied three balancing constraints: (1) comparable representation of abstract and figurative styles, (2) approximately equal distribution across high-, medium-, and low-fluency levels based on the Visual Fluency Score, and (3) sufficient stylistic diversity. These 94 images effectively passed a visual “Turing test” and were selected as the official stimuli for Studies 1, 2, and 3, ensuring that any differences in aesthetic evaluation observed in the main experiments could be confidently attributed to the cognitive impact of the creator label rather than to perceptible visual quality differences.

## 5 Study 1

Study 1 tested whether people exhibit negative bias toward AI-generated art and whether this effect is not attributable solely to the artworks themselves (Hypothesis 1). Participants viewed pairs of paintings. Although all artworks were in fact AI-generated, creator identity was manipulated by labeling one as human-created and the other as AI-generated; this manipulation was counterbalanced across participants. Participants then rated their subjective liking for each artwork, allowing us to examine whether the label alone led to differences in evaluation.

### 5.1 Method

A total of 67 participants were recruited for this study, with 65 valid datasets obtained after excluding 2 participants who did not complete all experimental trials (15 male, 23.08%, mean age 24). Sensitivity power analysis for paired-sample t-tests was conducted using G*Power. With an alpha level of 0.05 and power of 80%, the current sample size allowed detection of a minimum effect size of dz. = 0.35.

The study employed a mixed counterbalanced labeling design. At the participant level, both AI and human labels were presented within subjects on each trial; at the item level, creator labels were counterbalanced across participants so that each painting appeared equally often under both labeling conditions. To control for content effects, creator labels were fully counterbalanced: each painting was labeled as “human-created” for one group of participants and as “AI-generated” for another group. This ensured that each artwork was evaluated equally under both labeling conditions.

The study was conducted in a controlled laboratory environment. Participants were informed that the study aimed to evaluate paintings from different sources. A total of 40 paintings, covering a range of abstract and figurative styles, were used as stimuli. Paintings presented in pairs within each trial were carefully matched to ensure similarity in both subject matter and style. This ensured that within-pair variance in liking ratings was not confounded by differences in content or style.

The experiment consisted of 20 trials. In each trial, two paintings were presented side by side: one labeled “human-created” and the other “AI-generated.” This paired, simultaneous presentation design was deliberately chosen to maximize the salience of the creator identity labels. As previous research indicates, negative bias toward AI-generated art is often particularly pronounced under conditions of direct comparison with ostensibly human-made works (Horton et al., 2023). However, we acknowledge that this forced-comparison format is an influential design feature that likely contributes to the magnitude of the observed effect. After viewing each pair, participants rated their subjective liking for each painting using a 7-point Likert scale (1 = very low liking, 7 = very high liking). Participants provided independent ratings for the left and right images on the screen.

### 5.2 Results

To appropriately account for the nested structure of the data (multiple ratings nested within both participants and items), we analyzed the trial-level data using a linear mixed-effects model (LMM). The model specified liking ratings as the dependent variable, with creator label (AI vs. human) and representational style (figurative vs. abstract) entered as fixed-effect predictors along with their interaction. Random intercepts for both participants and items (artworks) were included to account for by-subject and by-stimulus variability. Type III ANOVAs used Satterthwaite’s method for denominator degrees of freedom, while *post-hoc* contrasts and simple-effects analyses via the emmeans package used Kenward-Roger degrees of freedom.

#### 5.2.1 Main effect of creator label

The main effect of creator label was significant, *F*(1, 2494.13) = 113.82, *p* < 0.001. Artworks labeled as “AI-generated” (estimated marginal mean *M* = 4.87, SE = 0.10, 95% CI [4.66, 5.07]) received significantly lower liking ratings than the same artworks labeled as “human-created” (*M* = 5.29, SE = 0.10, 95% CI [5.09, 5.49]), with an estimated difference of −0.424, SE = 0.040, *t*(2494) = −10.67, *p* < 0.001, confirming Hypothesis 1. The approximate Cohen’s d, computed using the residual standard deviation as the denominator, was −0.42, indicating a small-to-medium effect.

#### 5.2.2 Main effect of representational style

The main effect of representational style was significant, *F*(1, 38.00) = 47.24, *p* < 0.001. Figurative artworks (*M* = 5.50, SE = 0.11) were rated significantly higher than abstract artworks (*M* = 4.66, SE = 0.12), estimated difference = 0.835, SE = 0.122, *t*(38) = 6.87, *p* < 0.001.

#### 5.2.3 Interaction between creator label and representational style

The interaction between creator label and representational style was not significant, *F*(1, 2494.13) = 1.02, *p* = 0.314, indicating that the negative bias toward the AI label was consistent across both figurative and abstract artworks. Simple-effects analyses confirmed this pattern: the AI label significantly reduced aesthetic evaluations in both figurative artworks (Human − AI = 0.464, SE = 0.053, *t*(2494) = 8.70, *p* < 0.001) and abstract artworks (Human − AI = 0.384, SE = 0.059, *t*(2494) = 6.51, *p* < 0.001).

#### 5.2.4 Random effects

Analyses of the random effects indicated significant variability across both participants (by-participant random-intercept variance = 0.414, SD = 0.643) and items (by-item random-intercept variance = 0.131, SD = 0.361), with a residual variance of 1.016 (SD = 1.008). The presence of substantial between-participant and between-stimulus variability underscores the importance of using a mixed-effects approach. After accounting for these random sources of variability, the creator label continued to exert a significant negative effect on aesthetic evaluation that was consistent across representational styles, providing robust support for Hypothesis 1.

## 6 Study 2

Study 2 aimed to experimentally separate the automatic processing and controlled processing stages of aesthetic experience to examine the cognitive mechanisms underlying bias toward AI-generated art.

According to the PIA model, aesthetic preference can arise from stimulus-driven automatic processing, which generates aesthetic pleasure, or from perceiver-driven controlled processing, in which disfluency reduction produces aesthetic interest. We hypothesized that the negative impact of an AI label on aesthetic evaluation is processing-stage dependent. Specifically, bias is much stronger under conditions designed to favor controlled processing than under conditions designed to constrain it.

### 6.1 Method

A total of 126 college students participated in the study (69 male, 54.76%, mean age 22), receiving compensation for their involvement. Sensitivity power analysis using G*Power with *α* = 0.05 and 80% power indicated that the current sample size could detect a minimum effect size of *f* = 0.125 (ηp^2^ = 0.015).

The study was conducted in a controlled laboratory setting. Thirty paintings representing a range of abstract and figurative styles were selected as stimuli. The experiment employed a 2 (processing mode: automatic vs. controlled) × 2 (creator label: AI vs. human) mixed design, with processing mode as a between-subjects factor and creator label as a within-subjects factor. All paintings were presented in a counterbalanced order, and the creator label (AI vs. human) was manipulated accordingly.

To ensure that participants attended to the creator label, a forced-attention mechanism was implemented. Before each trial, the label of the painting was displayed on the screen, accompanied by two clickable buttons labeled “Human” and “AI.” Participants had to click the button corresponding to the label before the painting was shown. This procedure minimized the possibility of participants ignoring the label, ensuring the validity of the creator label manipulation.

According to the PIA model, the initiation of controlled processing depends on both processing ability and processing motivation. To effectively separate the two processing stages, we manipulated cognitive load (to limit processing ability) and need for cognitive enrichment (to manipulate processing motivation) in combination.

#### 6.1.1 Automatic processing group (cognitive resource-limited condition)

This group was designed to block high-resource, controlled processing, limiting aesthetic experience to the initial, stimulus-driven stage.

According to the PIA model, automatic processing is mandatory and default, requiring minimal cognitive resources. When cognitive ability is limited, participants cannot perform active cognitive reconstruction. Their aesthetic judgments rely only on fluency discrepancies and the resulting emotional response. Ratings in this condition were intended to rely more heavily on immediate perceptual impressions and less on extended cognitive elaboration.

Participants in this condition completed aesthetic evaluations under a dual-task paradigm. At the start of each trial, a randomly generated four-digit number was displayed. Participants were instructed to mentally rehearse and retain the number while viewing the painting, continuously occupying their working memory. After confirming the creator label by clicking the corresponding button, the painting was presented for 500 ms, followed immediately by a mask to remove any afterimage and prevent further cognitive processing. Participants then rated their overall liking of the painting on a 7-point Likert scale within 2.5 s. They were explicitly told: “Please rely on your intuition and first impression to quickly rate the visual appeal of the painting. Do not overthink or analyze.” After rating, participants had to correctly enter the four-digit number. Only trials in which the number was entered correctly were recorded for analysis. This ensured that cognitive resources were effectively occupied throughout the task.

#### 6.1.2 Controlled processing group (cognitive reflection-promoting condition)

This group was designed to activate perceiver-driven processing, encouraging participants to engage in active meaning construction and detailed cognitive elaboration with the artworks.

According to the PIA model, the initiation of controlled processing depends not only on cognitive ability, but also on an individual’s need for cognitive enrichment. This need drives participants to move beyond initial impressions and actively elaborate on the stimulus, updating their knowledge structures to make sense of the artwork. Under this mode, aesthetic evaluation depends on whether participants experience disfluency reduction during elaboration, which is directly related to aesthetic interest.

No secondary cognitive tasks were imposed, ensuring that participants’ cognitive resources were fully available. After confirming the creator label by pressing the corresponding button, participants were given ample time to view each painting (at least 5 s). They then rated the painting’s overall liking on a 7-point Likert scale. Participants were explicitly instructed: “Do not rate based solely on your first impression. Before evaluating, carefully observe the painting, actively consider the intended creative meaning, visual form, and deeper significance, and then provide your rating.” This manipulation was designed to stimulate a high level of need for cognitive enrichment, shifting the aesthetic experience from pleasure toward the exploration of meaning and interest.

### 6.2 Results

In the automatic processing condition, participants correctly recalled the four-digit number on 96.04% of trials on average; only trials with correct recall were included in the analyses.

To appropriately account for the nested structure of the data (multiple ratings nested within both participants and items), we analyzed the trial-level data using a linear mixed-effects model (LMM). The model specified liking ratings as the dependent variable, with processing mode (automatic vs. controlled), creator label (AI vs. human), and representational style (figurative vs. abstract) as fixed-effect predictors, including all two- and three-way interactions. Random intercepts for participants and items (artworks) were included to account for by-subject and by-stimulus variability. Type III ANOVAs on fixed effects used Satterthwaite’s method for denominator degrees of freedom. All *post-hoc* contrasts and simple-effects analyses were conducted using estimated marginal means via the emmeans package, with degrees of freedom computed using the Kenward-Roger approximation, which provides more accurate standard errors and degrees of freedom for contrasts in linear mixed-effects models (Luke, 2017).

#### 6.2.1 Main effect of creator label

The main effect of creator label was significant, *F*(1, 3568.8) = 58.01, *p* < 0.001. Artworks labeled as “AI-generated” (estimated marginal mean *M* = 5.03, SE = 0.08, 95% CI [4.87, 5.19]) received significantly lower liking ratings than those labeled as “human-created” (*M* = 5.31, SE = 0.08, 95% CI [5.15, 5.47]), estimated difference = −0.282, SE = 0.037, *t*(3569) = −7.61, *p* < 0.001.

#### 6.2.2 Main effect of processing mode

The main effect of processing mode was significant, *F*(1, 124.2) = 6.66, *p* = 0.011. Overall ratings were higher in the automatic processing condition (*M* = 5.33, SE = 0.10) than in the controlled processing condition (*M* = 5.01, SE = 0.10), estimated difference = 0.315, SE = 0.122, *t*(124) = 2.58, *p* = 0.011. This pattern can be understood within the PIA framework: in the automatic processing condition, evaluations were based primarily on immediate perceptual fluency, whereas in the controlled processing condition participants were instructed to move beyond first impressions and actively engage in meaning construction. This shift raises the evaluative threshold—ratings no longer depend solely on perceptual fluency but additionally require successful disfluency reduction. When this deeper elaboration does not yield a satisfying sense of understanding, ratings tend to be lower than those driven by immediate perceptual pleasure alone. This finding is consistent with the well-documented observation in empirical aesthetics that deliberative evaluation often produces more moderate or critical ratings than intuitive judgment (Wilson and Schooler, 1991), and with the PIA model’s prediction that controlled processing carries the inherent risk of processing failure, which can result in aesthetic confusion and lower evaluations.

#### 6.2.3 Main effect of representational style

The main effect of representational style was significant, *F*(1, 28.0) = 26.67, *p* < 0.001. Figurative artworks (*M* = 5.44, SE = 0.09) were rated significantly higher than abstract artworks (*M* = 4.90, SE = 0.10), estimated difference = 0.539, SE = 0.104, *t*(28) = 5.17, *p* < 0.001.

Critical interaction: processing mode × creator label. Crucially, the interaction between processing mode and creator label was significant, *F*(1, 3545.4) = 21.77, *p* < 0.001, providing evidence that the bias against AI-generated art emerges primarily during perceiver-driven controlled processing (H2).

#### 6.2.4 Simple-effects analyses revealed the source of this interaction

##### 6.2.4.1 Automatic processing condition

**Figure 3:**
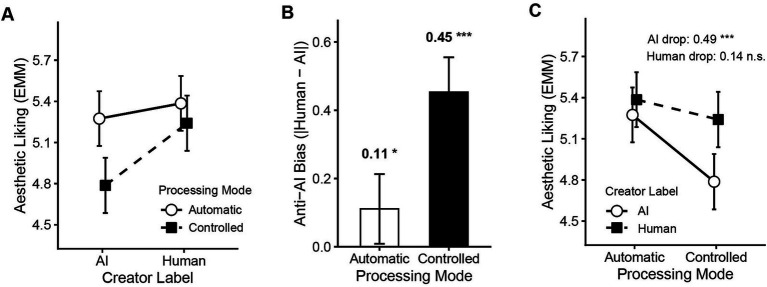
Results of study 2: Processing mode moderates anti-AI bias. **(A)** Estimated marginal means (EMMs) of aesthetic liking by creator label and processing mode. **(B)** The magnitude of anti-AI bias (|Human − AI|) was approximately four times larger under controlled than automatic processing. **(C)** The shift from automatic to controlled processing selectively reduced ratings for AI-labeled artworks. Error bars represent 95% CIs. ^*^p < 0.05, ***p < 0.001.

Under high cognitive load, the difference between human-labeled artworks (*M* = 5.39, SE = 0.10) and AI-labeled artworks (*M* = 5.27, SE = 0.10) was small, estimated difference = −0.112, SE = 0.052, *t*(3557) = −2.16, *p* = 0.031 (approximate Cohen’s d = −0.10) (see Figure 3).

##### 6.2.4.2 Controlled processing condition

When deep meaning construction was encouraged, human-labeled artworks (*M* = 5.24, SE = 0.10) were rated substantially higher than AI-labeled artworks (*M* = 4.79, SE = 0.10), estimated difference = −0.452, SE = 0.052, *t*(3562) = −8.70, *p* < 0.001 (approximate Cohen’s d = −0.41).

Although the AI-versus-human difference was statistically significant in both conditions, the magnitude of the bias was approximately four times larger under controlled processing than under automatic processing (0.452 vs. 0.112), consistent with our theoretical prediction (H2).

##### 6.2.4.3 Higher-order interactions involving style

To examine whether representational style further moderated these patterns, we additionally examined all higher-order interactions involving style. Neither the label × style interaction, *F*(1, 3580.6) = 0.11, *p* = 0.735, the group × style interaction, *F*(1, 3545.0) = 0.48, *p* = 0.487, nor the three-way interaction, *F*(1, 3556.5) = 1.11, *p* = 0.291, was significant. This pattern confirms that the bias is broadly consistent across representational formats under controlled processing.

##### 6.2.4.4 Random effects

Analyses of the random effects indicated that the by-participant random-intercept variance was 0.426 (SD = 0.653), the by-item random-intercept variance was 0.071 (SD = 0.267), and the residual variance was 1.220 (SD = 1.105). All variance components were substantial, justifying the use of a mixed-effects framework.

After accounting for these sources of variation, the processing mode × creator label interaction remained significant, providing robust support for Hypothesis 2: the negative bias against AI-generated art is markedly stronger under controlled processing than under automatic processing.

According to the PIA model, high ratings under controlled processing—reflecting aesthetic interest—depend on successful disfluency reduction. One interpretation is that for human-labeled artworks, deep processing enhances fluency through inferring artistic intention, whereas for AI-labeled artworks, viewers invest cognitive effort but, presuming an absence of genuine intention, fail to achieve this “aha” experience. The resulting state of “cognitive effort without fluency gain” produces aesthetic confusion and a significant devaluation of AI artworks.

## 7 Study 3

The results of Study 2 indicate that negative evaluations of AI-generated art do not arise solely from perceptual aversion during automatic processing, but primarily occur during the perceiver-driven controlled processing stage. According to the PIA model, when participants experience a high need for cognitive enrichment but lack the necessary contextual support, they cannot obtain the expected cognitive reward from the stimulus that is, they fail to achieve disfluency reduction. This leads to aesthetic confusion, lowering overall aesthetic ratings. For AI-generated art, viewers often fail to perceive creative intention through controlled processing, resulting in a cognitive impasse of “attempted meaning construction but failure.” Therefore, Study 3 aimed to explore a cognitively based intervention strategy: providing interpretive semantic cues to support meaning construction and enhance the elaboration affordance during the interaction between participant and artwork. We hypothesized that supplying context that explains the work’s intention or theme would help participants successfully achieve dynamic disfluency reduction during controlled processing. This, in turn, would generate aesthetic interest arising from a feeling of cognitive mastery, ultimately reducing negative bias toward AI art. Additionally, this study separately measured aesthetic pleasure and aesthetic interest to test whether interest, as a product specific to controlled processing, mediates the reduction of bias.

### 7.1 Method

Sensitivity power analysis for the 2 (creator label: AI vs. human, within-subjects) × 2 (semantic elaboration: present vs. absent, between-subjects) mixed ANOVA was conducted using G*Power. With an alpha level of 0.05 and power of 80%, the current sample size (*N* = 127; intervention group *N* = 67, control group *N* = 60) allowed detection of a minimum effect size of *f* = 0.124 (ηp^2^ = 0.015) for the between-subjects main effect and the within-between interaction, and *f* = 0.088 (ηp^2^ = 0.008) for the within-subjects main effect of creator label.

The study employed a 2 (creator label: AI vs. human) × 2 (interpretive semantic cues: present vs. absent) mixed design, with creator label as a within-subjects factor and cue availability as a between-subjects factor. Participants were randomly assigned to either the No-Cue Condition (*N* = 60) or the Semantic-Cue Condition (*N* = 67).

A total of 24 paintings of varying styles were used as stimuli. All paintings were presented in counterbalanced order, and creator labels (AI vs. human) were manipulated across trials.

#### 7.1.1 No-cue condition

Paintings were presented together with their creator label (“AI-generated” or “human artist”), with no additional information provided.

#### 7.1.2 Semantic-cue condition

In this condition, each painting was presented with its creator label and an interpretive semantic cue consisting of approximately 30 words. To ensure neutrality with respect to creator identity, all descriptions were written in a phenomenological style that focused exclusively on observable visual properties and potential symbolic meanings. Descriptions systematically avoided agentive language referencing any creator (e.g., “the artist intended,” “the model generates,” “carefully crafted”) and instead employed neutral constructions centered on the artwork itself (e.g., “the work presents,” “forms suggest,” “visual elements evoke”). Identical descriptions were used regardless of whether the painting was labeled as AI-generated or human-created.

Each description followed a standardized structure: (1) an objective identification of formal features, (2) an open-ended interpretive possibility, and (3) a prompt encouraging deeper engagement. For example: “A figure sits alone in the snowy forest, with dim lights in the distant town. The muted grays and bright flecks may evoke stillness or longing. Notice how the textured marks shapes the atmosphere.”

All texts were generated by GPT-4 using prompts specifying the neutral phenomenological approach, then manually reviewed and edited to remove any identity-revealing language. Descriptions were controlled for length, readability, and information density, and contained no value judgments, aesthetic evaluations, or references to artistic status.

According to the PIA model, when facing high-disfluency AI artworks, such interpretive semantic cues can enhance the participant’s coping potential, helping them successfully achieve disfluency reduction. This process aims to prevent controlled processing from failing during meaning construction, which would otherwise lead to aesthetic confusion, and instead foster positive aesthetic interest.

After viewing each painting along with its associated information (with a minimum viewing time of 5 s to ensure controlled processing), participants rated the artwork on a 7-point Likert scale for three questions, corresponding to overall preference and the dual-path outcomes proposed by the PIA model.

Aesthetic Liking: “Overall, how much do you like this artwork?” (1 = dislike very much, 7 = like very much). This serves as the measure of overall aesthetic evaluation.

Aesthetic Pleasure: “Does your first impression of this artwork make you feel pleasant and comfortable?” (1 = not at all, 7 = very pleasant). According to the PIA model, pleasure primarily reflects fluency-based, immediate emotional responses and does not involve deep reasoning.

Aesthetic Interest: “Do you find this artwork interesting? Would you like to learn more about it?” (1 = boring/uninteresting, 7 = interesting/want to explore). According to the PIA model, interest reflects the positive experience arising from successful disfluency reduction through cognitive effort and represents a forward-looking exploratory motivation.

### 7.2 Results

To appropriately account for the nested structure of the data (multiple ratings nested within both participants and items), we analyzed the trial-level data using linear mixed-effects models (LMMs) for the main analyses, and multilevel Bayesian mediation models (via the bmlm package in R) for the mechanism analyses. For each of the three dependent variables (overall aesthetic liking, aesthetic pleasure, and aesthetic interest), we fitted a model specifying the rating as the dependent variable, with cue condition (no-cue vs. semantic-cue), creator label (AI vs. human), and representational style (figurative vs. abstract) as fixed-effect predictors, including all two- and three-way interactions. Random intercepts for participants and items (artworks) were included to account for by-subject and by-stimulus variability. Type III ANOVAs used Satterthwaite’s method for denominator degrees of freedom, while *post-hoc* contrasts and simple-effects analyses conducted via the emmeans package used the Kenward-Roger approximation, which provides more accurate estimates for complex contrast structures.

#### 7.2.1 Overall aesthetic liking

The main effect of creator label was significant, *F*(1, 2905.21) = 42.36, *p* < 0.001. Artworks labeled as “AI-generated” (estimated marginal mean *M* = 5.03, SE = 0.09, 95% CI [4.85, 5.21]) received significantly lower liking ratings than those labeled as “human-created” (*M* = 5.29, SE = 0.09, 95% CI [5.11, 5.47]), estimated difference = −0.260, SE = 0.040, *t*(2905) = −6.51, *p* < 0.001. The main effect of cue condition was also significant, *F*(1, 124.97) = 7.13, *p* = 0.009, with higher ratings in the semantic-cue condition (*M* = 5.35, SE = 0.11) than in the no-cue condition (*M* = 4.97, SE = 0.12), estimated difference = 0.375, SE = 0.140, *t*(125) = 2.67, *p* = 0.009. The main effect of representational style was significant, *F*(1, 22.02) = 23.92, *p* < 0.001, with figurative artworks (*M* = 5.45, SE = 0.11) rated higher than abstract artworks (*M* = 4.87, SE = 0.11), estimated difference = 0.582, SE = 0.119, *t*(22) = 4.89, *p* < 0.001.

**Figure 4:**
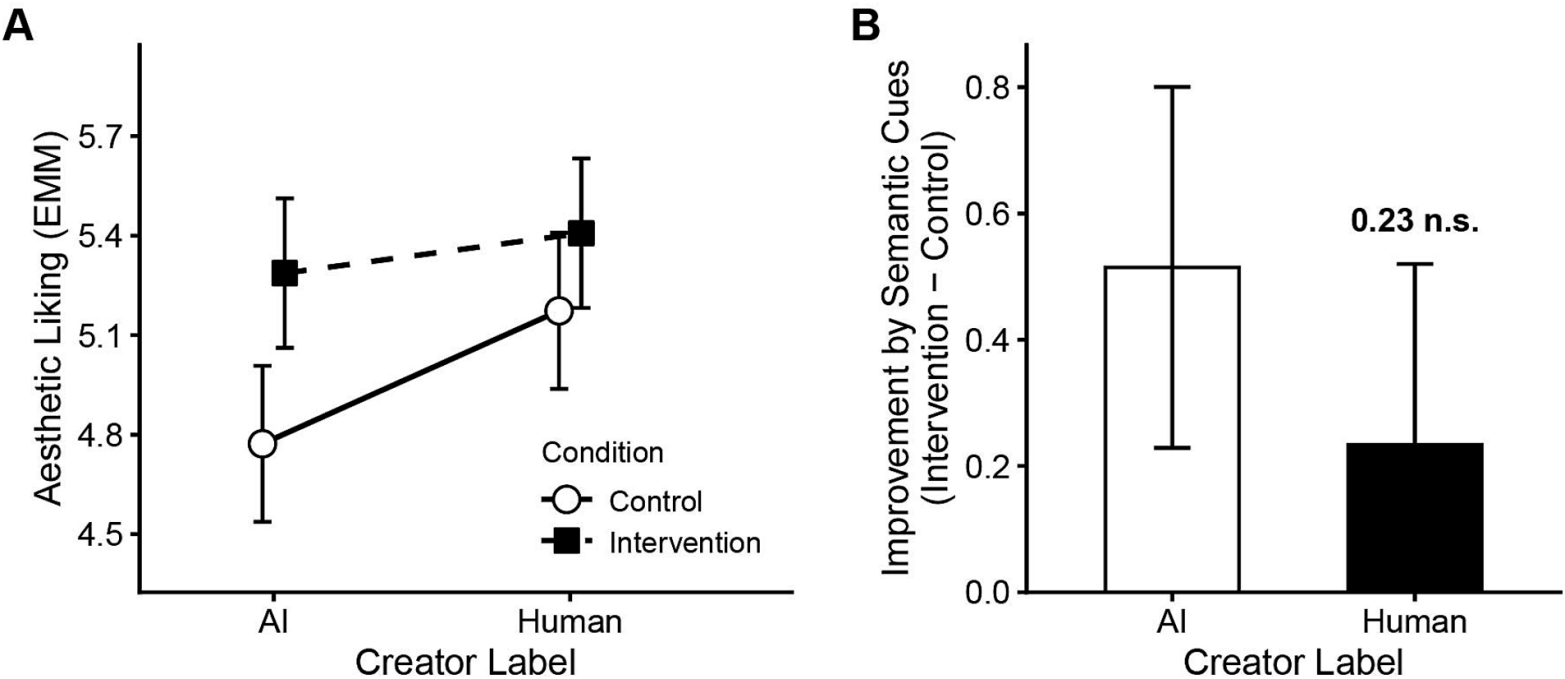
Results of study 3: Interpretive semantic cues attenuate anti-AI bias. **(A)** EMMs of aesthetic liking by creator label and condition. The anti-AI bias was substantially reduced in the intervention condition. **(B)** The improvement produced by semantic cues was larger for AI-labeled than for human-labeled artworks. Error bars represent 95% CIs.

Crucially, the cue condition × creator label interaction was significant, (Figure 4) *F*(1, 2894.41) = 12.45, *p* < 0.001, supporting H3 that interpretive semantic cues attenuate anti-AI bias. Simple-effects analyses revealed the source of this interaction. In the no-cue condition, participants showed a strong negative bias against AI art: AI-labeled artworks (estimated marginal mean *M* = 4.77, SE = 0.12) were rated significantly lower than human-labeled artworks (*M* = 5.17, SE = 0.12), estimated difference = −0.400, SE = 0.058, *t*(2903) = −6.90, *p* < 0.001. In the semantic-cue condition, this bias was substantially reduced: AI-labeled artworks (*M* = 5.29, SE = 0.12) and human-labeled artworks (*M* = 5.41, SE = 0.12) differed by only −0.120, SE = 0.055, *t*(2897) = −2.21, *p* = 0.027. Although the AI-versus-human difference remained statistically significant in both conditions, the bias in the no-cue condition was more than three times larger than that in the semantic-cue condition (0.40 vs. 0.12). Importantly, the cue intervention primarily benefited AI-labeled artworks: the effect of interpretive semantic cues on liking was substantially larger for AI-labeled artworks (*Δ* = 0.515, SE = 0.146, *t*(146) = 3.53, *p* < 0.001) than for human-labeled artworks (Δ = 0.235, SE = 0.146, *t*(146) = 1.61, *p* = 0.109).

The condition × style interaction, *F*(1, 2892.97) = 7.96, *p* = 0.005, and the label × style interaction, *F*(1, 2918.24) = 5.54, *p* = 0.019, were significant, whereas the three-way interaction was not, *F*(1, 2906.93) = 1.34, *p* = 0.247. The non-significant three-way interaction indicates that the attenuation of anti-AI bias by semantic cues was broadly consistent across representational formats. Random-effects variances were all substantial (by-subject = 0.575, by-ite*M* = 0.076, residual = 1.187), justifying the use of a mixed-effects framework.

#### 7.2.2 Aesthetic pleasure

**Figure 5:**
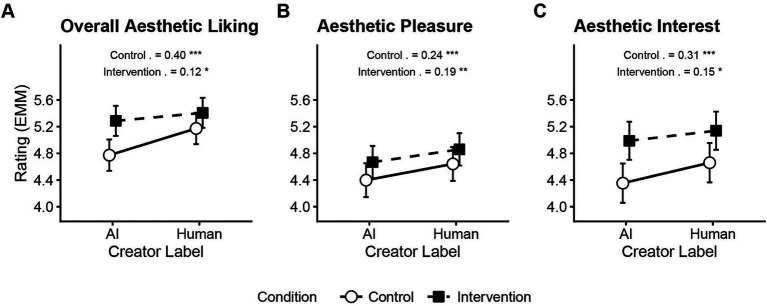
Dissociation across aesthetic liking, pleasure, and interest (Study 3). EMMs for **(A)** overall aesthetic liking, **(B)** aesthetic pleasure, and **(C)** aesthetic interest, as a function of creator label and condition. Semantic cues primarily elevated ratings for liking and interest, but had little impact on pleasure, consistent with the PIA model’s prediction. Error bars represent 95% CIs. *p < 0.05, **p < 0.01, ***p < 0.001.

The main effect of creator label was significant, (see Figure 5B), *F*(1, 2905.95) = 25.15, *p* < 0.001, with AI-labeled artworks rated lower than human-labeled artworks in both the no-cue (Δ = −0.244, SE = 0.063, *t*(2904) = −3.86, *p* < 0.001) and semantic-cue (*Δ* = −0.193, SE = 0.059, *t*(2897) = −3.24, *p* = 0.001) conditions. The main effect of representational style was significant, *F*(1, 22.02) = 38.51, *p* < 0.001, with figurative artworks rated as more pleasing than abstract artworks. Critically, neither the main effect of cue condition, *F*(1, 124.95) = 2.55, *p* = 0.113, nor the condition × creator label interaction, *F*(1, 2894.51) = 0.35, *p* = 0.552, was significant. These results indicate that semantic cues did not significantly alter immediate perceptual pleasure toward AI artworks.

#### 7.2.3 Aesthetic interest

The main effect of creator label was significant, (see Figure 5C) *F*(1, 2898.97) = 27.32, *p* < 0.001, with AI-labeled artworks (*M* = 4.67, SE = 0.13) rated as less interesting than human-labeled artworks (*M* = 4.90, SE = 0.13). Importantly, the main effect of cue condition was highly significant, *F*(1, 124.95) = 13.09, *p* < 0.001, indicating that semantic cues substantially enhanced aesthetic interest (no-cue: *M* = 4.51, SE = 0.15; semantic-cue: *M* = 5.06, SE = 0.14), estimated difference = 0.558, SE = 0.154, *t*(125) = 3.62, *p* < 0.001. The condition × creator label interaction on interest was not statistically significant, *F*(1, 2893.59) = 3.20, *p* = 0.074. Descriptively, the AI-versus-human difference in interest ratings was numerically larger in the no-cue condition (*Δ* = −0.306, SE = 0.064, *t*(2898) = −4.83, *p* < 0.001) than in the semantic-cue condition (Δ = −0.151, SE = 0.060, *t*(2895) = −2.54, *p* = 0.011), but this differential attenuation did not reach conventional significance levels. Importantly, H3 concerns bias reduction in overall liking (demonstrated by the significant condition × label interaction on liking, *p* < 0.001), while interest serves as the theoretical mediator rather than requiring a significant interaction itself.

#### 7.2.4 Multilevel mediation analyses

**Figure 6:**
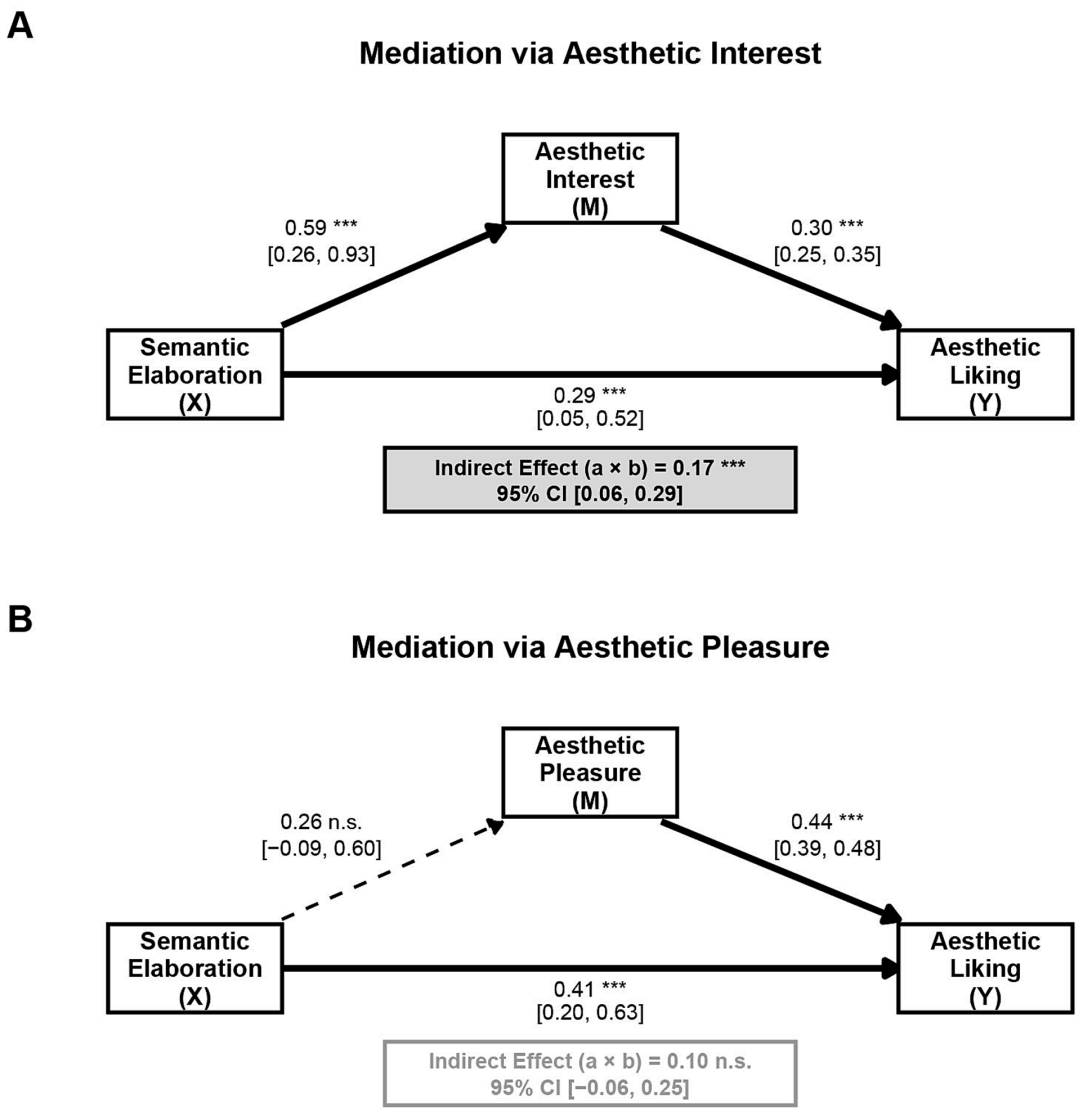
Multilevel Bayesian Mediation Models (Study 3, AI-labeled trials only). **(A)** Mediation via aesthetic interest: the indirect effect was significant (ab = 0.17, 95% CI [0.06, 0.29]). **(B)** Mediation via aesthetic pleasure: the indirect effect was not significant (ab = 0.10, 95% CI [−0.06, 0.25]). Values represent posterior means with 95% CIs. Dashed arrow indicates a non-significant path. ***p < .001 denotes 95% CI excluding zero; n.s. = 95% CI includes zero.

To test the PIA-based hypothesis that interpretive semantic cues reduce anti-AI bias specifically through aesthetic interest rather than aesthetic pleasure, we conducted multilevel Bayesian mediation analyses using the bmlm package, which partitions within- and between-person variance and uses Bayesian estimation to yield full posterior distributions of direct, indirect, and total effects with 95% credible intervals (CIs). Analyses were restricted to trials with AI-labeled artworks (*N* = 1,524 observations from 127 participants), because the theoretical question concerns whether semantic cues specifically affect liking for AI art. Two parallel mediation models were fitted with 4,000 iterations across 4 chains: (see Figure 6) (1) cue condition → aesthetic interest → aesthetic liking; and (2) cue condition → aesthetic pleasure → aesthetic liking. All Bayesian models showed acceptable convergence (Rˆ = 1.00 for all parameters).

#### 7.2.5 Mediation via aesthetic interest

All paths in this model were credibly non-zero (i.e., the 95% CIs excluded zero): the a path from condition to interest (a = 0.594, SE = 0.170, 95% CI [0.265, 0.938]), the b path from interest to liking (b = 0.298, SE = 0.025, 95% CI [0.250, 0.347]), the direct effect (c’ = 0.293, SE = 0.120, 95% CI [0.060, 0.533]), and the total effect (c = 0.465, SE = 0.159, 95% CI [0.158, 0.781]). Critically, the indirect effect was credibly non-zero, ab = 0.173, SE = 0.059, 95% CI [0.060, 0.293], P(ab > 0) = 0.999, indicating that interpretive semantic cues significantly enhanced liking for AI artworks by increasing aesthetic interest.

#### 7.2.6 Mediation via aesthetic pleasure

The direct effect (c’ = 0.409, SE = 0.110, 95% CI [0.194, 0.624]), the b path from pleasure to liking (b = 0.436, SE = 0.023, 95% CI [0.389, 0.481]), and the total effect (c = 0.511, SE = 0.153, 95% CI [0.210, 0.813]) were all credibly non-zero. However, the a path from condition to pleasure was not credibly different from zero (a = 0.259, SE = 0.170, 95% CI [−0.075, 0.596]), and consequently the indirect effect through aesthetic pleasure was not credibly non-zero, ab = 0.102, SE = 0.078, 95% CI [−0.052, 0.255], P(ab > 0) = 0.907. Thus, although aesthetic pleasure strongly predicted liking at the trial level, interpretive semantic cues did not significantly increase pleasure, and pleasure therefore did not carry the experimental effect.

#### 7.2.7 Comparing the two mediators

The posterior distribution of the difference between the two indirect effects (interest ab − pleasure ab) had a mean of 0.070 (SE = 0.098, 95% CI [−0.119, 0.265]), with P(interest ab > pleasure ab) = 0.768. Although the two indirect effects did not differ credibly from each other, the pattern of results clearly distinguishes their roles: only the interest pathway yielded a credibly non-zero indirect effect, whereas the pleasure pathway did not. This dissociation supports the PIA-based prediction (H3) that reducing bias against AI art operates through meaning construction during controlled processing (aesthetic interest) rather than through immediate sensory pleasure.

Taken together, the LMM and multilevel mediation results converge to show that providing interpretive semantic cues that facilitate meaning construction substantially attenuates anti-AI bias, and that this attenuation is carried by aesthetic interest—a hallmark of successful disfluency reduction during controlled processing—rather than by aesthetic pleasure, which reflects stimulus-driven automatic processing.

## 8 General discussion

Across three progressive experiments, this study examined the psychological mechanisms underlying systematic devaluation of AI-generated art. The results show that this aversion does not stem from visual or sensory rejection, but from cognitive disruption. Specifically, although AI artworks are visually comparable to human-made works, the label “AI-generated” acts as a cognitive barrier during the controlled processing stage. Notably, the bias was substantially larger under controlled processing (mean difference = 0.45) than under automatic processing (mean difference = 0.11), confirming that this bias is primarily rooted in controlled processing rather than in perceptual aversion. Our findings thus reveal the cognitive—rather than sensory—nature of this bias (Study 2). Importantly, this bias is not irreversible. By providing interpretive semantic cues that support meaning construction, we successfully endowed the artworks with elaboration affordance and, through the pathway of enhanced aesthetic interest, significantly reduced devaluation of AI art (Study 3). Building on these empirical findings, we offer a more speculative mechanistic interpretation grounded in the PIA model (Graf and Landwehr, 2015). We suggest that the main barrier to acceptance of AI-generated art may lie in a cognitive impasse during controlled processing, potentially caused by a failure of disfluency reduction when viewers are unable to effectively construct meaning—perhaps because the “AI-generated” label undermines assumptions about creative intention behind the work. Although neither processing disfluency nor perceived creative intention was directly measured in the present studies, this interpretation is consistent with the overall pattern of results: the bias emerged selectively under controlled processing conditions and was alleviated when semantic cues were provided to facilitate cognitive elaboration. Future research that directly measures or manipulates these constructs would be valuable for testing this account more rigorously.

This study applies the PIA model to the fields of human-computer interaction and art psychology, offering a unique theoretical contribution to understanding aesthetic experience in the algorithmic era. First, it extends the classical boundaries of processing fluency theory. Traditional fluency theory emphasizes a single path “ease of processing equals beauty” (Reber et al., 2004). Our results, however, support Graf and Landwehr’s (2015) central claim that aesthetic experience involves a dual-level pathway: a stimulus-driven shallow pleasure and a perceiver-driven deeper interest. In the context of AI art, we identified a distinctive “fluency-meaning mismatch” phenomenon. AI artworks often exhibit high perceptual fluency due to statistical regularities, which makes them pleasant under automatic processing. Yet, because they lack recognizable human creative intent, they fail to satisfy the Need for Cognitive Enrichment during controlled processing. This demonstrates that aesthetic evaluation depends not only on the physical properties of the object but also on whether the perceiver can confirm their own cognitive coping potential while interacting with it (Silvia, 2005).

Beyond its theoretical significance, this study offers practical implications for the curation, dissemination, and human-AI collaborative creation of art. First, for the art market and museums, simple “blind tests” or emphasizing only the visual appeal of AI artworks (i.e., targeting aesthetic pleasure) may be insufficient to overcome public resistance. Our findings suggest that fostering aesthetic interest is key. This implies that AI art displays require a stronger contextual support system than human art such as detailed creation backgrounds, metaphorical explanations of algorithmic logic, or narratives of human-AI collaboration to provide sufficient elaboration affordance. Second, for generative AI developers and users, these results highlight the role of explainability at the aesthetic level. If viewers can understand the logic behind AI-generated images or the design intent of prompts, this understanding can itself serve as a source of disfluency reduction, transforming potential aesthetic confusion into positive aesthetic interest. Finally, reducing this bias is not only about promoting technology it is about enriching human aesthetic experience. As the PIA model suggests, by overcoming cognitive resistance to novel stimuli and achieving moments of insight, people can gain more enduring and profound satisfaction than from mere sensory pleasure alone.

Although this study provides strong evidence, several limitations remain, which also suggest directions for future research.

First, we did not examine how antecedents of the Need for Cognitive Enrichment (NFCE) might moderate AI art acceptance. According to Graf and Landwehr (2015), NFCE can arise from both personal traits (e.g., openness to experience) and situational factors. The participants in this study were predominantly young adults (mean age 22–25), resulting in a relatively homogeneous sample in terms of age and background. Differences across age groups in technology familiarity, art experience, and cognitive processing styles may all influence aesthetic responses to AI art. Future research could recruit participants with a broader age range and more diverse cultural and educational backgrounds to enhance the external validity of the aesthetic cognition findings.

Second, regarding stimulus materials, this study focused primarily on static paintings. This setup represents a conservative test: if a brief textual description can significantly alter viewers’ deep aesthetic evaluation of a static image, then in more complex interactive AI art or dynamic generative media, the role of meaning-making support is likely to be even stronger.

Additionally, although we controlled for visual quality, the label “AI-generated” may trigger different social representations across cultural contexts (e.g., fear of automation or reverence for technology). Future cross-cultural studies could clarify the boundary conditions of this effect.

By applying the PIA model, we demonstrate that as long as an interpretive bridge is provided helping viewers traverse the cognitive path from confusion to insight AI art can fully evoke deep aesthetic interest. This suggests that in a human-AI symbiotic future, the generation of artistic value will depend less on who creates the work and more on how we collaboratively construct and understand its meaning.

## Data Availability

The datasets presented in this study can be found in online repositories. The names of the repository/repositories and accession number(s) can be found at: https://zenodo.org/records/20328268.
